# Multimodal Magnetic Resonance and Near-Infrared-Fluorescent Imaging of Intraperitoneal Ovarian Cancer Using a Dual-Mode-Dual-Gadolinium Liposomal Contrast Agent

**DOI:** 10.1038/srep38991

**Published:** 2016-12-22

**Authors:** M. K. Ravoori, S. Singh, R. Bhavane, A. K. Sood, B. Anvari, J. Bankson, A. Annapragada, V. Kundra

**Affiliations:** 1 Department of Cancer Systems Imaging, U.T.-M.D. Anderson Cancer Center, 1400 Pressler St., Unit 1473, Houston, TX 77030, USA; 2 Department of Pediatric Radiology, Texas Children’s Hospital, 6621 Fannin St., Houston, TX 77030, USA; 3 Department of Gynecologic Oncology, U.T.-M.D. Anderson Cancer Center, 1400 Pressler St., Houston, TX 77030, USA; 4 Department of Bioengineering, University of California, Riverside, 900 University Ave., 211 Materials Science & Engineering Building, Riverside, California, USA; 5 Department of Imaging Physics, U.T.-M.D. Anderson Cancer Center, 1400 Pressler St., Houston, TX 77030, USA; 6 Department of Radiology, U.T.-M.D. Anderson Cancer Center, 1400 Pressler St., Houston, TX 77030, USA.

## Abstract

The degree of tumor removal at surgery is a major factor in predicting outcome for ovarian cancer. A single multimodality agent that can be used with magnetic resonance (MR) for staging and pre-surgical planning, and with optical imaging to aid surgical removal of tumors, would present a new paradigm for ovarian cancer. We assessed whether a dual-mode, dual-Gadolinium (DM-Dual-Gd-ICG) contrast agent can be used to visualize ovarian tumors in the peritoneal cavity by multimodal MR and near infra-red imaging (NIR). Intraperitoneal ovarian tumors (Hey-A8 or OVCAR3) in mice enhanced on MR two days after intravenous DM-Dual Gd-ICG injection compared to controls (SNR, CNR, p < 0.05, n = 6). As seen on open abdomen and excised tumors views and confirmed by optical radiant efficiency measurement, Hey-A8 or OVCAR3 tumors from animals injected with DM-Dual Gd-ICG had increased fluorescence (p < 0.05, n = 6). This suggests clinical potential to localize ovarian tumors by MR for staging and surgical planning, and, by NIR at surgery for resection.

Ovarian cancer is the most lethal gynecologic malignancy^1^. Nearly 75% of patients are first diagnosed with tumor within the peritoneal cavity (intraperitoneal disease), portending poor survival^2^. Surgery is the frontline intervention. Locating and characterizing tumor implants is critically important for staging and pre-surgical planning. Clinically, CT or MR + traditional extravascular-extracellular contrast is used for staging and pre-surgical planning, but are limited in sensitivity for tumor detection and in distinguishing benign from malignant disease^3^,^4^,^5^,^6^. Moreover, tumor enhancement is only temporary on the order of minutes, which is acceptable for rapid diagnostic tests but not if one also wants to mark tumors for surgical resection days later. Locating tumor implants and then removing them at the time of surgery is important for outcome. In fact, the degree of cytoreduction, i.e. tumor resection, at surgery is one of the most important factors predicting overall survival, with improved survival associated with complete resection of all visible cancer^7^,^8^. Practically, this commonly translates into removing all >1–2 cm lesions visible by the naked eye^9^. Detecting even these in the large space of the peritoneum is challenging. A multimodality agent that can be used for MR for staging and pre-surgical planning and for optical imaging to aid surgical removal of ovarian cancer would present a new paradigm in clinical imaging of ovarian cancer.

MR has greater soft-tissue contrast than CT and does not use ionizing radiation. Traditional extravascular-extracellular contrast agents enhance lesions for only a few minutes. The dual-Gd platform with both surface and encapsulated Gd has greater relaxivity than only surface Gd or only encapsulated Gd liposomes, and has approximately 10,000 times greater relaxivity/particle than traditional extravascular-extracellular agents and has been shown to increase SNR, for example, for vascular imaging^10^. Because of its diameter (≈100–250 nm), it should extravasate via enhanced-permeability-and-retention-effect (EPR). This occurs in nearly all solid tumors but not normal tissues and is attributable to abnormal angiogenic vessels in tumors. Moreover, similar liposomes are retained in tumors for 2–3 days or longer^11^.

Among optical imaging techniques, NIR affords low background and greater depth of penetration than visible light^12^,^13^,^14^. This real-time technique has been proposed for use during surgery^15^,^16^. Among NIR agents, only ICG is clinically approved, primarily for eye angiography^17^. Free ICG has a short plasma half-life (~2–4 minutes)^18^,^19^. Encapsulation increases circulation time and delays maximal hepatic accumulation (>1 hour)^20^. Encapsulation can also increase *in vivo* stability, and signal intensity^21^.

Clinically, imaging agents are needed that can detect and characterize peritoneal lesions as metastases. A high relaxivity nanoparticle with dual modality MR and NIR capability and long term retention in tumor may also find clinical utility for first pre-surgical planning and then lesion detection at surgery. The purpose of this work was to assess whether a dual mode, dual gadolinium liposomal contrast agent (DM-Dual-Gd-ICG, Fig. 1) can be used to visualize intraperitoneal ovarian tumors by multimodal MR and NIR imaging.

## Results

### Liposomes

Five batches of liposomes were prepared and characterized (Table 1). All of the batches exhibited nearly equivalent mean diameter and polydispersity index. The cumulative size distributions of all batches showed that 100% of the particles were between 100 and 200 nm, most <150 nm. Gd, P, and ICG levels in all batches were similar. Phosphorus levels indicated a total lipid concentration of ~100 mM, compared to the nominal value of 150 mM estimated from the starting lipid amounts, suggesting ~33% loss of lipids during the extrusion process, consistent with previous experience.

**Figure 2:**
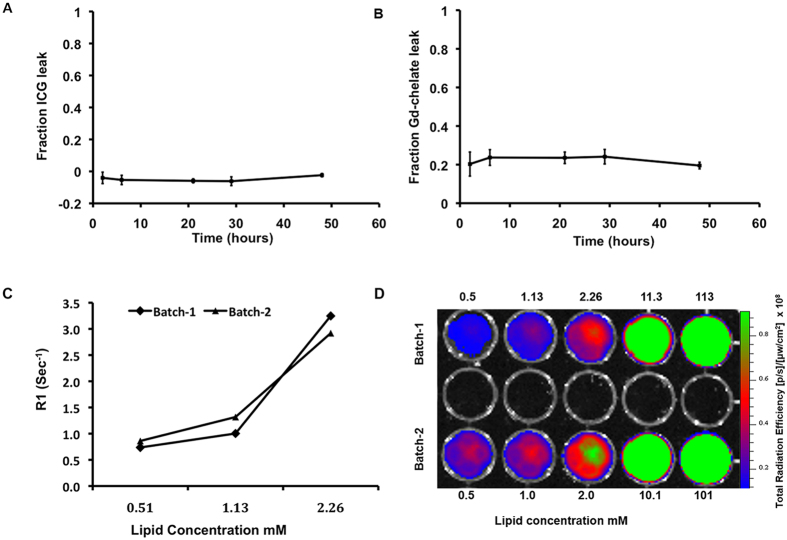
*In vitro* leak study of liposomes in plasma at 37 °C for 48 hours. (**A**) ICG and (**B**) Gd-chelate in triplicate. T_1_ relaxation rate (R1) (**C**) and total radiant efficiency (**D**) of two batches of DM-Dual-Gd-ICG are similar. Dilutions of different batches of DM-Dual-Gd-ICG were measured by MR (**C**) or by NIRF (**D**) imaging.

ICG leak from the particles over time at 37 °C in plasma was undetectable (Fig. 2A); an initial ~20% leak of Gd-chelate was noted at 2 hours when this plateaued and no further leak was seen (Fig. 2B). T_1_ relaxation rates (R1) (Fig. 2C) and total radiant efficiency (Fig. 2D) of the two different batches used for animal experiments (batch-1 and batch-2) were similar. Thus, there was good batch to batch reproducibility.

### MR and NIRF imaging

**Figure 3:**
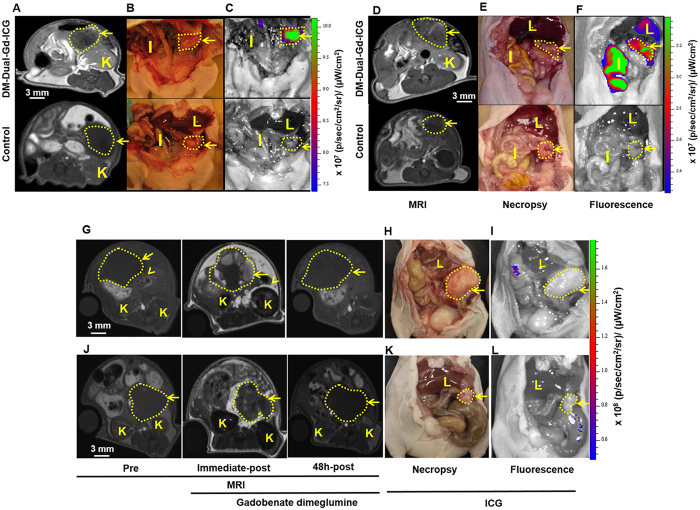
*In vivo* MR imaging and NIRF imaging shows that DM-Dual-Gd-ICG results in enhancement of HeyA8 tumors 2 days later. Representative axial 2D-FSPGR MR images of mice injected IV with DM-Dual-Gd-ICG (top) or vehicle (bottom) (**A**). Representative coronal necropsy (**B**) and coronal open abdomen near infrared fluorescence images (**C**) of a nude mice. *In vivo* MR imaging and NIR imaging shows that DM-Dual-Gd-ICG liposomes results in enhancement of OVCAR-3 tumors 2 days later. Representative axial 2D-FSPGR MR images of mice injected IV with DM-Dual-Gd-ICG (top) or vehicle (bottom) (**D**). Representative coronal necropsy (**E**) and coronal open abdomen near infrared fluorescence images (**F**) of a nude mice. Representative MR and NIR imaging show that free ICG or gadobenate dimeglumine do not result in increased signal in HeyA8 tumors 2 days after IV injection. Representative axial 2D-FSPGR MR images pre, immediate-post and 2 days after IV injection of gadobenate dimeglumine (**G**). Representative coronal necropsy (**H**) and coronal open abdomen near infrared fluorescence images (**I**) of nude mice. Representative MR and NIR imaging show that free ICG or gadobenate dimeglumine do not result in increased signal in OVCAR3 tumors 2 days after IV injection. Representative axial 2D-FSPGR MR images pre, immediate-post and 2 days-post after IV injection of gadobenate dimeglumine (**J**). Representative coronal necropsy (**K**) and coronal open abdomen near infrared flouresence images (**L**) of a nude mice. Arrow, tumor; I, intestine; K, kidney; L, Liver.

**Figure 4:**
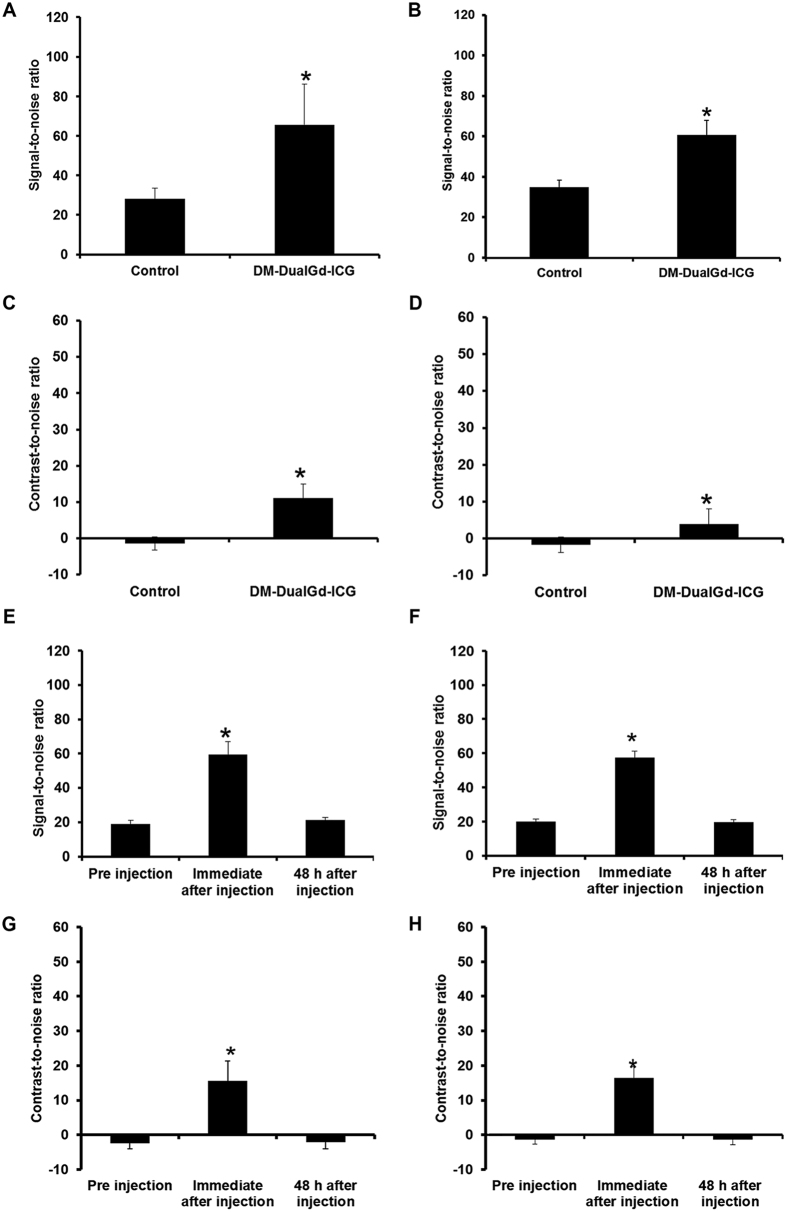
Quantitative assessment of signal-to-noise ratio of DM-DualGd-ICG uptake in intraperitoneal (**A**) HeyA8 tumors and (**B**) OVCAR3 tumors and contrast-to-noise ratio of DM-DualGd-ICG uptake in intraperitoneal (**C**) HeyA8 tumors and (**D**) OVCAR3 tumors 48 hours after injection using MRI. *P < 0.05; n = 6 Hey-A8, n = 5 OVCAR3. Quantitative assessment of signal-to-noise ratio pre, immediate-post and 2 days after IV gadobenate dimeglumine in (**E**) HeyA8 tumors and (**F**) OVCAR3 tumors and contrast-to-noise ratio pre, immediate-post and 2 days after IV gadobenate dimeglumine in (**G**) HeyA8 tumors and (**H**) OVCAR3 tumors. *P < 0.05; n = 6 Hey-A8, n = 6 OVCAR3.

Intraperitoneal tumors were established using human ovarian cancer HeyA8 cells or OVCAR-3 cells. Two days after injection, HeyA8 tumors from mice injected with DM-Dual-Gd-ICG had greater enhancement compared to tumors from control mice as seen by *in vivo* MR (Fig. 3A). The mice were then sacrificed and immediately, optical NIR imaging followed, which again demonstrated increased signal in HeyA8 tumors from mice injected with DM-Dual-Gd-ICG compared to tumors from control mice (Fig. 3B and C). Similar MR and NIR findings were noted with a second intraperitoneal tumor model, OVCAR-3 (Fig. 3D–F). Reticuloendothelial system (RES) uptake in the liver and spleen is expected with liposomal agents; and, via hepatobilliary excretion, signal in the bowel (and with reflux, in the stomach) is expected and would move in time due to peristalsis in a live animal. Physiologically, the degree of excretion and peristalsis is expected to be variable between individual mice. To determine if leakage of encapsulated ICG or MR contrast agent from liposomes resulted in findings at the 48 hour time point, *in vivo* studies were performed using mice bearing intraperitoneal HeyA8 or OVCAR3 tumors. For robustness, non-encapsulated ICG or gadobenate dimeglumine was injected at the full dose expected to be delivered with DM-Dual-Gd-ICG liposomes, as if the liposomes leaked 100% of their payload. Immediately after injection, HeyA8 tumors from mice injected with gadobenate dimeglumine enhanced compared to the same animal tumors before injection as expected but not two days after injection (Fig. 3G). Likewise, two days after injection, no signal was seen in HeyA8 tumors by NIR imaging (3H and I). Similar MR and NIR findings were noted with the OVCAR3 model (Fig. 3J,K and L), thus, the signal seen at 48 hours (Fig. 3A–F) was due to the liposome formulation. The MR signal-to-noise ratio and contrast-to-noise ratio were significantly greater in HeyA8 tumors from mice injected with DM-Dual-Gd-ICG than tumors from control mice (p < 0.001 SNR, p < 0.01 CNR, n = 6, Fig. 4A and C). Similar findings were noted with the second intraperitoneal tumor model, OVCAR-3 (p < 0.01 SNR, p < 0.001 CNR n = 5, Fig. 4B and D). Differences in the degree of signal are expected when using two different tumor models. In control studies, signal-to-noise and contrast-to-noise MR ratios were increased in HeyA8 tumors immediately after iv injection with gadobenate dimeglumine (p < 0.001, n = 6, Fig. 4E and G) compared to before injection as expected, but not two days after injection; no difference in signal was seen two days after injection versus before injection. Similar findings were noted with the OVCAR3 model (p < 0.001, n = 6, Fig. 4F and H). Significantly, in both models, increased T_1_-weighted MR signal could be visualized in intraperitoneal tumors in mice two days after DM-Dual-Gd-ICG injection.

### *Ex vivo* NIRF Imaging

**Figure 5:**
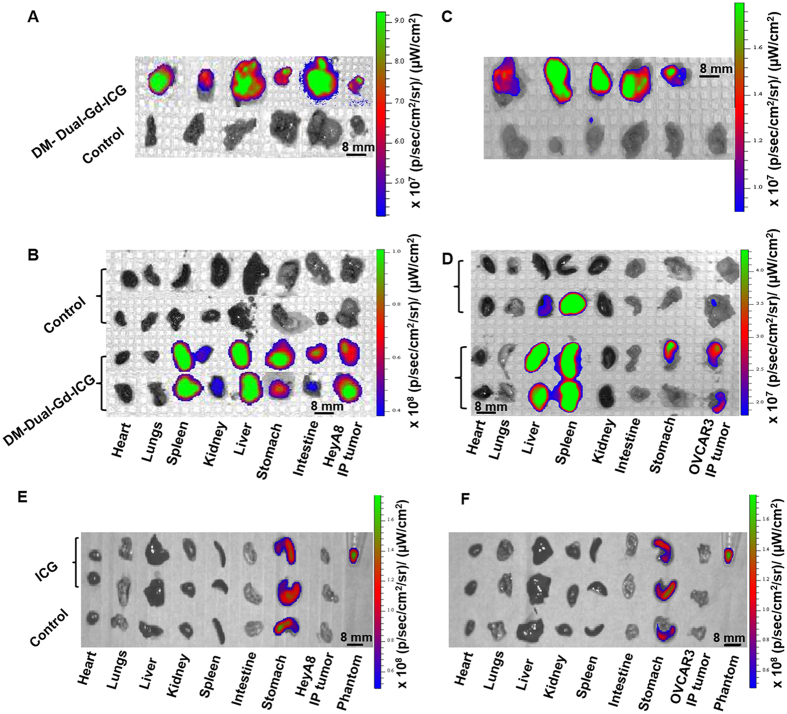
Optical imaging and *ex vivo* organ distribution of DM-Dual-Gd-ICG in HeyA8 and OVCAR-3 tumor-bearing mice 2 days after injection. Near infrared fluorescence images of DM-Dual-Gd-ICG uptake by intraperitoneal HeyA8 tumors (**A**) and different organs (**B**). Near infrared fluorescence images of DM-Dual-Gd-ICG uptake by intraperitoneal OVCAR-3 tumors (**C**) and different organs (**D**). Near infrared fluorescence images of ICG uptake by intraperitoneal (**E**) HeyA8 tumors or (**F**) OVCAR3 tumors and different organs.

**Figure 6:**
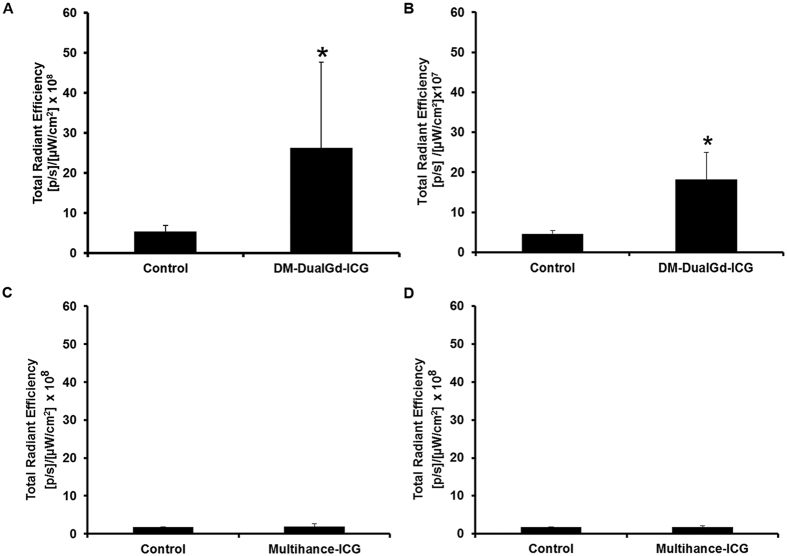
Semi-quantitative evaluation of near infrared fluorescence by HeyA8 tumors (**A**, *P < 0.05, n = 6) or OVCAR3 tumors (**B**, *P < 0.05, n = 5) 2 days after mice were injected with DM-Dual-Gd-ICG or vehicle. Semi-quantitative evaluation of near infrared fluorescence by HeyA8 tumors (**C**, n = 6) or OVCAR3 tumors (**D**, n = 6) from mice injected with ICG.

Immediately after whole body optical NIR imaging, *ex-vivo* imaging of tumors and organs was performed. HeyA8 tumors from mice injected with DM-Dual-Gd-ICG had greater signal compared to tumors from control mice (Fig. 5A) whereas mice injected with ICG had no increased signal (Fig. 5E). As expected with DM-Dual-Gd-ICG, signal was also seen in extracted organs such as the liver and spleen (Fig. 5B). In addition, hepatobilliary excreted material is expected in the intestines and, via reflux, in the stomach (Fig. 5B). Similar findings were noted with the OVCAR-3 model (Fig. 5C,D and F). Semi-quantitative evaluation of fluorescence intensity in intraperitoneal HeyA8 tumors showed significantly greater signal in the DM-Dual-Gd-ICG group compared to the control group (p < 0.04, n = 6, Fig. 6A). No increased signal was seen in the ICG group (Fig. 6C). Similar findings were noted with a second intraperitoneal tumor model, OVCAR-3 (p < 0.001, Fig. 6B and D). There was some variability in the degree of uptake of DM-Dual-Gd-ICG by the tumors, as has been reported with other liposomal formulations previously. Differences in the degree of signal are expected when using two different tumor models. Significantly, in both models, increased NIR signal could be visualized in intraperitoneal tumors in mice two days after DM-Dual-Gd-ICG injection.

## Discussion

This is the first demonstration of dual mode MR and NIR imaging of tumors using a single positive contrast nanoparticle in peritoneal human ovarian cancer models. The agent has long term residence in ovarian tumors, therefore, after a single injection, it enables MR imaging followed by optical NIR imaging. In addition to lesion detection and characterization, there is potential for MR imaging for pre-surgical planning followed by NIR imaging at the time of surgery for lesion detection and resection.

Dual mode imaging can be advantageous compared to single mode imaging. Strengths of MR include exquisite display of anatomy enabling an understanding of signal from a nanoparticle in context of anatomic surroundings, percutaneous imaging *in vivo* that is generally not depth limited, and resolution on the order of millimeters clinically; however, MR machines are not used during abdominal surgery. Because signal detection can be limited in MR, amplification at the level of the nanoparticle, such as afforded by the Dual-Gd approach, is needed. Strengths of optical imaging include enabling dynamic imaging in the real time of surgery which should allow lesion detection and confirmation of resection, portable imaging systems that may be used in the operating room environment, fluorescence and white light imaging to enhance visualization of the lesion and surrounding eloquent anatomy, and spatial resolution at the <100 nm scale^22^. NIR imaging enables better depth of penetration (1–2 cm) than visible light imaging, however, this still is inadequate for percutaneous imaging for tumor staging and treatment planning. The dual mode nanoparticle design includes external and internal Gd-chelates for MR imaging, ICG for NIR imaging, and PEGylation to avoid RES uptake. It enables EPR effect for tumor localization; increased duration of MR and optical signal in the tumor compared to standard extravascular-extracellular MR agents or high dose ICG alone; signal amplification; long residence time to enable pre-surgical planning and surgery, and, to potentially improve clinical workflow efficiency for MR by providing a long window between nanoparticle injection and MR imaging. We applied DM-Dual Gd-ICG to ovarian cancer models.

Ovarian cancer is most commonly diagnosed at an advanced stage with approximately three-quarters of patients presenting with peritoneal implants at the time of diagnosis^2^. Imaging of patients with advanced disease is usually performed using CT, MR, or PET/CT. Detection is limited by implant size as well as location and reports comparing percutaneous imaging modalities have had mixed findings regarding the superiority of any one test^4^,^5^,^23^,^24^,^25^,^26^,^27^,^28^. Both PET and CT utilize ionizing radiation, whereas, MR does not making it potentially safer. Mechanistically, both CT and MR intravenous contrast materials used for ovarian tumor imaging are extravascular-extracellular agents. Ricke *et al*. noted that conventional intravenous MR contrast agents can improve sensitivity, but sensitivity is location dependent and varied from 38% to 83% in locations such as omentum, lesser sac, bladder, bowel, mesentery and lower pelvis^6^. Thus, conventional extra-vascular-extracellular contrast agents have limited utility for detection. In addition, they enhance tumors for a short duration on the order of a few minutes but not days, as we confirmed, limiting their utility for multipurpose (detection, surgical planning, and intraoperative imaging) applications.

Due to abnormal angiogenic and lymphatic vessels, most solid tumors exhibit enhanced permeability and retention (EPR) effect^29^, enabling nanoparticle localization and extended residence. We reasoned that the long residence time of DM-Dual-Gd-ICG could be advantageous by allowing MR and then later optical imaging. Indeed, DM-Dual-Gd-ICG was visible by MR two days after injection and could be imaged by optical imaging afterwards. Liposomes with a similar lipid composition as DM-Dual-Gd-ICG have been shown to be retained in tumors for more than five days^11^. Practically, such wide time windows would afford sufficient time for pre-surgical MR imaging followed by NIR imaging at surgery.

Liposomes are subject to reticuloendothelial system (RES) uptake in the liver and spleen. In order to limit this and extend circulation time, the liposomes were coated with polyethylene glycol (PEG). Physiologically, the liposomal nanoparticle is expected to be removed from the body via hepatobilliary excretion into in the duodenum from where it may reflux into the stomach and will travel through the bowel. Distinguishing signal from nanoparticles in the lumen of bowel is aided by peristalsis, which results in movement of nanoparticles in the lumen in a living subject. This concept is commonly used clinically to understand if signal is arising from material in the bowel.

Gadolinium based contrast agents cause T1-shortening, resulting in positive image contrast. DM-Dual-Gd-ICG is based on the dual-Gd platform meaning that Gd is found on both the surface and within the liposome. This increases Gd/particle resulting in greater signal/particle than particles with Gd only on the surface or only encapsulated, and results in approximately 10,000 times greater relaxivity/particle than traditional extravascular-extracellular contrast agents^10^.

Incorporation of ICG enabled optical imaging, which has numerous advantages such as high sensitivity and spatial resolution, real-time high frame rate imaging, relatively low cost, portability, and lack of ionizing radiation exposure^20^. ICG fluoresces in the near infrared wavelengths, where there is negligible autofluorescence as well as limited scatter and absorption in the body. This results in low background and greater tissue penetration to enable deeper optical imaging. At high doses (approximately 10 fold greater amount than the encapsulated material we delivered), intravenously delivered free ICG alone can result in optical signal in ovarian tumors, theoretically by behaving as a macromolecule via binding serum proteins like albumin, but signal in tumor is lost with time, such as 24 versus 6 hours after injection^30^. We did not find free ICG to result in increased signal in tumors at 48 hours at doses equivalent to DM-Dual-Gd-ICG, so free ICG binding serum proteins was not the mechanism of increased signal in tumor, rather it was due to ICG bound in DM-Dual-Gd-ICG. Free ICG has a short plasma half-life (~2–4 minutes)^18^,^19^. Encapsulation increases circulation time and delays maximal hepatic accumulation^20^. Encapsulation can also increase *in vivo* stability, and signal intensity^21^. A few mouse studies support the concept of fluorescent imaging of peritoneal ovarian tumor^30^,^31^,^32^,^33^,^34^,^35^. These do not include an MR component and are generally targeted to one biomarker, limiting their utility to subsets of ovarian tumors. Satpathy *et al*. reported on iron oxide nanoparticles bound to a HER-2 antibody labeled with NIR-830^36^. This is interesting work. Limitations to iron oxide nanoparticles are that they result in more difficult to see negative contrast and generally are not currently used in the clinic. The targeted particles detected only some of the evaluated ovarian cell lines^36^. Mechanistically, nanoparticles such as DM-Dual-Gd-ICG localize to tumors via enhanced permeability and retention, which is not biomarker restricted. Variability in uptake of lipid-based nanoparticles by tumors, even by tumors derived from a single cell line, has been noted^11^. There was expected variability in MR and NIR signal in OVCAR-3 and HeyA8 derived tumors, but in all tumors, uptake was found; thus, variability in uptake did not prevent tumor detection in two different ovarian tumor models, suggesting that imaging based on DM-Dual-Gd-ICG is robust. Production of DM-dual-Gd-ICG was reproducible with five different batches demonstrating similar characteristics. Although this paper evaluated ovarian tumor models, the imaging methods described herein should also be applicable to other solid cancers.

For optical imaging, only two fluorophores (ICG and fluorescein) are FDA approved and between these, ICG is the only approved NIR fluorophore^37^. The components of DM-Dual-Gd-ICG have been used clinically, including ICG, liposomes, and gadolinium chelates. MR machines are readily available and for the operating room environment, NIR cameras have been described^22^,^38^,^39^. Thus, there is potential for clinical translation.

DM-Dual-Gd-ICG was designed to serve as both a positive MR contrast agent and a NIR optical probe. In this study, we found that DM-Dual-Gd-ICG detected intraperitoneal implants in two different peritoneal human ovarian cancer models by two different modalities after a single injection. Although DM-Dual-Gd-ICG will require safety testing, the use of components found in FDA approved drugs to synthesize DM-Dual-Gd-ICG as well as the wide time window for imaging of days after administration should facilitates practical translation of this method. The current findings suggest clinical potential for using a single injection of a single nanoparticle (DM-Dual-Gd-ICG) to localize ovarian tumors by MR for pre-surgical planning and by NIR at the time of surgery to guide resection.

## Methods

### DM-Dual-Gd-ICG

1,2-Dipalmitoyl-sn-Glycero-3-Phosphatidylcholine (DPPC), cholesterol (Chol), and diethylenetriaminepentaacetic acid-bis (stearylamide) gadolinium salt (Gd-DTPA-BS) were purchased from Avanti Polar lipids (Alabaster, AL, USA). N-(carbonyl-methoxy polyethylene glycol 2000)-1,2-Distearoyl-sn-Glycero-3 phosphatidylethanolamine (mPEG-2000-DSPE) was purchased from Genzyme Pharmaceuticals (Cambridge, MA, USA). Indocyanine green (ICG) was obtained from Sigma-Aldrich (St. Louis, MO, USA). Whatman Nuclepore polycarbonate track-etch membranes of 100 and 400 nm pore sizes were purchased from Fisher Scientific (Waltham, MA, USA). Dual-Gd liposomes consisting of encapsulated and surface Gd with a nominal 150 mM lipid content and encapsulating ICG were made as follows.

Lipids consisting of 30:40:5:25 mole percent of DPPC:Chol:DSPE-mPEG-2000-DSPE:Gd-DTPA-BS were dissolved in ethanol at 65 °C. A 1.5 mM solution of ICG was made in deionized (DI) water. This was used to make a 65 μM solution of ICG in gadobenate dimeglumine (505 mg/ml, Bracco Diagnostics Inc., Princeton, NJ, USA). The lipids dissolved in ethanol were then hydrated with this solution to encapsulate the ICG and gadobenate dimeglumine using constant stirring for 30 minutes at 65 °C. Ethanol content was 10% by volume of the total suspension. The liposomes were then extruded 5 times through 400 nm followed by 6 extrusions through 100 nm Nucleopore membranes using a high-pressure extruder (Northern Lipids, Vancouver, BC, Canada). Extrusions were done at 65 °C.

The liposomal suspensions were diafiltered using a MicroKros module (Spectrum Laboratories Inc., CA, USA) of 500 kDa molecular weight cutoff (MWCO) membrane tubes to remove unencapsulated ICG and Gd (gadobenate dimeglumine). 10 mM histidine with 140 mM saline (pH~7.4) was used as the replacement buffer.

Total Gd and P (phospholipid) content of the liposomal formulations was determined by inductively-coupled plasma optical emission spectroscopy (ICP-OES, Model Optima 4300D, Perkin Elmer, Norwalk, CT, USA) operating at a wavelength of 336.223 nm for gadolinium, and 213.617 nm for phosphorus. The phosphorus levels were used to estimate the total lipid concentration, since the phospholipids are the only phosphorus-containing component of the formulation. Liposome particle size was determined using a BI-90 Brookhaven Instruments Dynamic Light Scattering instrument (Brookhaven Instruments Corp., Holtsville, NY, USA) using a 532 nm laser, 90° scattering angle and Hamamatsu photomultiplier. The correlation function was fitted using a cumulants analyses. The measurement was repeated on separate samples (N = 3) of each batch. The Z-average (mean) diameter and polydispersity were recorded for each measurement. The correlation function was also inverted using the CONTIN algorithm, and the intensity-weighted distribution of particle sizes that resulted was converted to a volume-weighted distribution using the Mie coefficients at the mid-point of each size interval in the histogram. Cumulative size distributions were calculated using the volume-weighted distributions, and the cumulative fractions below 100 nm, below 150 nm and below 200 nm were reported.

In order to study leakage of encapsulated Gd chelate and ICG from the liposomes (3 different batches used) in plasma at 37 °C over time, 100 μl of liposomes were incubated with 500 μl of bovine plasma at 37 °C for 2, 6, 21, 29, and 48 hours. After the incubation, the samples were diluted 30 times with histidine-saline and the liposomes were ultracentrifuged at 103, 250 × g for 1.25 hours at 18 °C using a Beckman L7-65 ultracentrifuge with a SW55 Ti rotor. The supernatant was collected and analyzed by ICP for Gd content, and by a lab-built NIR camera system for ICG content. No incubation was used as background and was subtracted from above. ICG was measured using a lab-built NIR camera system, consisting of led lights with a cutoff filter of 780 nm for the excitation of ICG. Images were acquired by a camera fitted with a 810–830 nm bandwidth filter (for ICG emission). ICG standards between 0.002–0.2 μM were made and images were acquired. A standard curve was generated by drawing a ROI (same dimension for all standards) in the cuvettes holding the standards and measuring the intensities. The same ROI was used to measure the samples.

T1 relaxation rate (R1) measurements of two different batches of DM-Dual-GD-ICG used for animal experiments were performed. Dilutions were imaged in a 4.7T MR (47/40 USR, Bruker Biospin, Billerica, MA). Quantitative T_1_ relaxation measurements were performed using a fast spin-echo saturation-recovery sequence (TR = 110–10000 ms [110 ms, 200 ms, 400 ms, 600 ms, 1000 ms, 2000 ms, 4000 ms, 6000 ms, 10000 ms]; TE = 50 ms; echo train = 8; field of view (FOV) = 4 cm × 3 cm; Image matrix = 128 × 128; number of signal averages = 1). T_1_-value was measured using a region of interest (ROI) encompassing each tube containing DM-Dual-Gd-ICG. Paravision version 4 was used to calculate the T_1_ relaxation values by exponentially fitting of signal as a function of TR. The relaxation rates (R1) of the diluted samples were obtained using the inverse of these values.

For assessing NIRF, two different batches of DM-Dual-Gd-ICG were individually diluted in saline. Samples were analyzed using a Xenogen IVIS 200 system (Perkin Elmer Inc., Waltham MA). Total radiant efficiency (p/s)/(μW/cm^2^) was determined using ICG (λ_excitation_ = 705–780 nm and λ_emission_ = 810- 885 nm) filters and drawing a region of interest (ROI) encompassing each well.

### Animals

Twelve female nude mice (6–8 weeks old) were acquired from the Experimental Radiation Oncology Department at UT- MD Anderson Cancer Center (Houston, TX) and housed in specific pathogen-free conditions. They were cared for under the guidelines set forth by the American Association for Accreditation of Laboratory Animal Care and all studies were approved and supervised by the MD Anderson Cancer Center Institutional Animal Care and Use Committee.

Tumors were established by intraperitoneal injection of 1 × 10^7^ HeyA8 or OVCAR-3 cells using a syringe with a 28 gauge needle. At weekly intervals after injection, the mice were monitored for tumor growth. After 3 week, mice (n = 6 per group) were randomly assigned to control group or experimental group. Then, the mice were injected intravenously with 200 μl of DM-Dual-Gd-ICG liposomes or vehicle and imaged after two days. Vehicle was chosen since clinically, the most appropriate control is enhancement pre vs post contrast agent imaging; and, in this manuscript we wanted to demonstrate the dual mode (MR and optical) nature of the nanoparticle.

As controls, mice (n = 6) bearing intraperitoneal HeyA8 or OVCAR3 tumors were injected intravenously with free (non-encapsulated) ICG or gadobenate dimeglumine, for robustness, delivered at the full dose delivered with DM-Dual-Gd-ICG. For MR, imaging was performed before, immediately after, and two days after intravenous injection. NIR imaging was performed two days after intravenous injection.

### Magnetic Resonance Imaging

All MR studies were performed using a 4.7 T scanner (Bruker Biospec, 47/40 USR, Bruker Biospin, Billerica, MA) with a 60 mm gradient insert and a volume resonator with a 35 mm inner diameter. Animals were anesthetized and placed head first and prone on a positioning sled. Orthogonal 3-plane scout scans were initially acquired to confirm animal positioning. Animal placement and tumor location were confirmed using images from a respiratory gated axial T2-weighted fast spin echo sequence, which essentially served as a localizer sequence (repetition time = 3813 ms, effective echo time (TE) = 57 ms, echo train = 12; field of view (FOV) = 4 × 3 × 4 cm, slice thickness = 1 mm, image matrix = 256 × 192, number of signal averages = 3, spatial resolution, 156 Am, flip angle = 180). For T1-weighted imaging, axial 2D fast spoiled gradient echo (2D-FSPGR) sequence (TR = 120.54 ms; TE = 1.50 ms; echo train = 1; field of view (FOV) = 3 × 3 × 4 cm; slice thickness = 1 mm; image matrix = 128 × 128; number of signal averages = 20, flip angle = 90) and coronal 3D fast spoiled gradient echo (3D-FSPGR) sequence (TR = 6.9 ms; TE = 1.56 ms; echo train = 1; field of view (FOV) = 3 × 5 cm; slice thickness = 0.625 mm; image matrix = 192 × 192; number of signal averages = 6, flip angle = 20) were acquired. The axial 2D fast spoiled gradient echo sequence was used to measure the signal intensity of the tumor tissue. Signal-to-noise (SNR) and contrast-to-noise (CNR) were calculated by manually drawn regions of interest (ROI) around the periphery of each slice containing tumor, muscle and background (air) using Bruker ParaVision 5.1 software. The SNR was measured as SI_*T*_/SD where, SI_*T*_ is the mean signal intensity of the tumor and SD is the standard deviation of the signal intensity of the background. The CNR was calculated as (SI_T_ − SI_m_)/SD, where SI_m_ is the mean signal intensity of the muscle. SNR and CNR were measured for each tumor and the average values were presented for each group.

### Near Infrared-Fluorescent Imaging

After MR imaging, the animals were sacrificed and immediately, NIR imaging of the open abdomen was performed. Tumors and organs (heart, lungs, liver, spleen, kidney, stomach, and intestine) were then dissected and excised, and fluorescence imaging of excised tumor and organs was performed. For NIR imaging, a Xenogen IVIS 200 system (Perkin Elmer Inc., Waltham, MA, USA) was used. ICG fluorophore excitation (λ_excitation_ = 705–780 nm) and emission (λ_emission_ = 810–885 nm) filter sets were used. Using Living Image 2.5 software, regions of interest (ROI) were drawn for each organ/tumor and total radiant efficiencies (p/s)/(μW/cm^2^) were measured.

### Statistical Analysis

For comparing groups, Student’s two-tailed t-tests were performed using spreadsheet software (Microsoft Office Excel, 2010; Seatle, WA). A p value of ≤0.05 was considered statistically significant.

## Additional Information

**How to cite this article**: Ravoori, M. K. *et al*. Multimodal Magnetic Resonance and Near-Infrared-Fluorescent Imaging of Intraperitoneal Ovarian Cancer Using a Dual-Mode-Dual-Gadolinium Liposomal Contrast Agent. *Sci. Rep.* **6**, 38991; doi: 10.1038/srep38991 (2016).

**Publisher's note:** Springer Nature remains neutral with regard to jurisdictional claims in published maps and institutional affiliations.

## Figures and Tables

**Figure 1:**
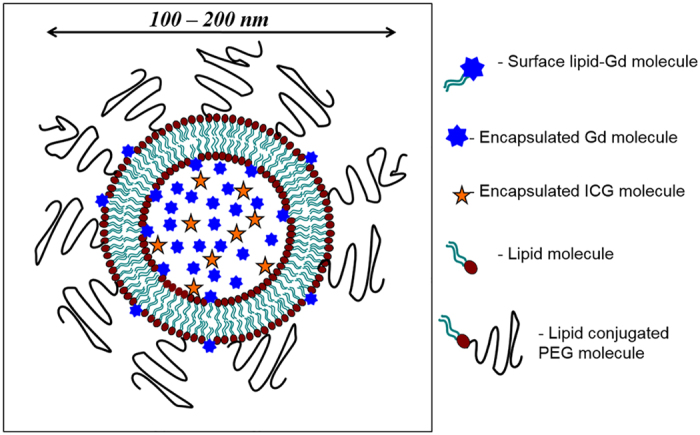
(**A**) Schematic of a Dual-Gd ICG liposome. It consists of a bilayer of lipid molecules encapsulating an aqueous interior of Gd-chelate and ICG molecules. The encapsulated Gd-chelate and the surface lipid-Gd-chelate provide contrast for MRI. The ICG is used in near-infrared imaging (NIR). The polyethylene glycol (PEG) conjugated to lipid increases the circulating time of the liposomes by avoiding reticuloendothelial cell system (RES) clearance.

**Table 1:** DM-Dual-Gd-ICG Batch Characteristics.

DM-Dual-Gd-ICG	Mean Diameter (nm)	Polydispersity Index	Cumulative Particle Size Distribution			Gd (mM)	P (mM)	Lipid (mM)	Estimated ICG (pM)
			% <200 nm	% <150 nm	% <100 nm				
Batch-1	135 +/− 11	0.08 +/− 0.03	100	95 +/− 9	0	66.50 +/− 1.52	39.60 +/− 0.65	113.00 +/− 1.86	49
Batch-2	137 +/− 20	0.06 +/− 0.07	100	67 +/− 58	0	66.60 +/− 0.11	35.4 +/− 0.26	101.20 +/− 0.76	44
Batch-3	136 +/− 4	0.06 +/− 0.05	100	100	0	75.57 +/− 0.36	36.34 +/− 0.55	103.81 +/− 1.56	45
Batch-4	155 +/− 2	0.04 +/− 0.03	100	62 +/− 13	0	84.73 +/− 1.20	35.78 +/− 0.35	102.21 +/− 1.00	44
Batch-5	147 +/− 1	0.01 +/− 0.00	100	99 +/− 2	0	78.71 +/− 4.76	36.78 +/− 0.18	105.10 +/− 0.51	46
